# Mercury Levels in an Urban Pregnant Population in Durham County, North Carolina

**DOI:** 10.3390/ijerph8030698

**Published:** 2011-03-01

**Authors:** Marie Lynn Miranda, Sharon Edwards, Pamela J. Maxson

**Affiliations:** Children’s Environmental Health Initiative, Nicholas School of the Environment, Duke University, Box 90328, Durham, NC 27708, USA; E-Mails: se@duke.edu (S.E.); pm12@duke.edu (P.J.M.)

**Keywords:** mercury, fish consumption, pregnant women

## Abstract

The adverse effects of prenatal mercury exposure, most commonly resulting from maternal fish consumption, have been detected at very low exposure levels. The omega-3 fatty acids found in fish, however, have been shown to support fetal brain and vision development. Using data from a prospective, cohort study of pregnant women from an inland area in the US South, we sought to understand the fish consumption habits and associated mercury levels across subpopulations. Over 30% of women had at least 1 μg/L of mercury in their blood, and about 2% had blood mercury levels above the level of concern during pregnancy (≥3.5 μg/L). Mercury levels were higher among Asian/Pacific Islander, older, higher educated, and married women. Fish consumption from any source was reported by 2/3 of the women in our study, with older women more likely to consume fish. Despite eating more fish meals per week, lower income, lower educated women had lower blood mercury levels than higher income, higher educated women. This suggests the different demographic groups consume different types of fish. Encouraging increased fish consumption while minimizing mercury exposure requires careful crafting of a complex health message.

## 1. Introduction

Fetal exposure to mercury can result in adverse developmental effects and is most commonly due to maternal fish consumption [1]. However, consuming omega-3 fatty acids, which is also associated with prenatal fish consumption, has positive effects on infant cognitive development [2–5]. These two contrasting considerations combine to present public health professionals and clinicians with the difficult task of delivering a complex environmental health message to women of childbearing age. For example, Oken *et al.*, in a cohort of women in Massachusetts, found that infants of women with the highest prenatal fish consumption but with low mercury levels had the highest infant and toddler cognition scores [3,4].

Fetal exposure to methylmercury, the biologically available form of mercury, has been associated with learning and memory deficits [6,7], as well as delayed cognitive performance [3,4]. Epidemiological birth cohort studies in the Faroe Islands indicate that prenatal mercury exposure leads to negative neurobehavioral effects in children, including reduced attention span, fine-motor function and verbal memory impairment [8,9], and mental and psychomotor retardation [10]. In a Polish study, prenatal maternal blood mercury levels greater than 0.50 μg/L raised the risk of delayed performance in children compared to prenatal maternal mercury levels less than 0.50 μg/L, with the risk for delayed performance increasing more than three-fold when cord blood mercury levels were greater than 0.80 μg/L [11]. Similar results were found in the World Trade Center cohort in New York City [12]. Prenatal mercury exposure has also been associated with preterm birth [13], cleft palate, hydrocephalus, and heart defects [14–16].

Fish consumption is typically recommended during pregnancy because of the positive neurodevelopmental effects associated with consuming omega-3 fatty acids [3,4,17–19], although some studies do not demonstrate a positive effect [20–23]. Research indicates that the omega-3 fatty acids found in seafood improve birth outcomes and support fetal brain and vision development [2]. Maternal prenatal fish consumption has been associated with increased fetal growth rates [17] and higher infant visual recognition memory at six months [3] and 3 years [4].

Balancing the benefits of maternal prenatal fish consumption against the risks of fetal exposure to mercury is particularly difficult as there may be no truly safe level of fetal mercury exposure. The US Environmental Protection Agency established a reference dose, the level at which adults should not experience adverse health effects, for blood mercury equivalent to 5.8 μg/L [24,25]. Evidence indicates that mercury concentrations in cord blood are significantly higher than those found in maternal blood [12,24,26], suggesting that transplacental transfer magnifies exposure levels. This has led some experts to suggest that a more appropriate safety threshold for women of childbearing age may be 3.5 μg/L in maternal blood [24,27]. Prenatal mercury exposures below this lower threshold have still been associated with preterm birth [13] and delays in neurological development [28], indicating that any prenatal exposure to mercury within the range that is measured in humans may pose a risk to fetal and child development.

Within the United States, racial, socioeconomic, and geographic differences in mercury levels and fish consumption have been reported [29]. The objective of this paper is to describe the blood mercury levels and demographic and fish consumption patterns in an urban pregnant population in a noncoastal area of the US South. We leverage data from North Carolina, which is an especially appropriate area for studying mercury because of the statewide mercury fish advisory for largemouth bass, a common recreational fishery. We examine differences in both fish consumption habits and blood mercury levels across demographic and socioeconomic subgroups. Through a consideration of the joint associations between maternal blood mercury levels, demographics, socioeconomic status, and dietary habits, we hope to provide the data required to help craft more effective public health messages around the complex issue of balancing the risks and benefits of fish consumption during pregnancy.

## 2. Methods

### 2.1. Study Population

The Healthy Pregnancy, Healthy Baby Study is an ongoing, prospective cohort study enrolling pregnant women living in Durham County, North Carolina, US, a county located roughly 150 miles inland from the Atlantic Coast. This study is a key component of the Southern Center on Environmentally-Driven Disparities in Birth Outcomes (SCEDDBO), an interdisciplinary center aimed at understanding how environmental, social, and host factors jointly contribute to health disparities.

Women receiving prenatal care at either the Duke Obstetrics Clinic or the Durham County Health Department Prenatal Clinic were eligible to participate if they planned to deliver at Duke University Medical Center, were at least 18 years of age, were English-literate, lived in Durham County, and did not have a multi-fetal gestation or any known fetal genetic or congenital anomalies. The Healthy Pregnancy, Healthy Baby Study and all associated analysis are conducted according to a research protocol approved by Duke’s Institutional Review Board.

### 2.2. Medical and Personal Data

We reviewed electronic medical records to collect detailed data on maternal medical and antepartum history, prenatal care, and neonatal outcomes. Demographic and health behavior information were collected through direct patient interview at the time of enrollment. Participants also completed surveys to assess socioeconomic status and dietary habits. As part of the dietary survey, participants were asked to report how many servings of fish, on average, they consumed per week from each of the following sources: canned tuna, store/restaurant-bought fish, and fish caught by themselves or a friend/family member.

### 2.3. Blood Mercury Levels

Maternal blood samples were collected at the time of hospital admission for delivery in a Monoject trace element blood collection tube containing EDTA as an anticoagulant. Samples were sent to Mayo Clinical Laboratories to be analyzed for total blood mercury using Inductively Coupled Plasma-Mass Spectometry. The detection limit of this method was 1 μg/L [30]. Because women vary in timing of delivery, blood samples were collected at anywhere from 23–42 weeks, with a median gestational age of 39 weeks and an interquartile range of 37–39 weeks. Only participants completing the study and for whom a valid blood mercury level was reported from Mayo Medical Laboratories were included in these analyses.

Blood mercury levels were reported as integer values, and over two-thirds of participants had mercury levels below the detection limit. We characterized maternal blood mercury levels into three ordered categories: below the detection limit (<1 μg/L), detectable but not elevated (1–3 μg/L), and elevated (≥4 μg/L). Blood mercury concentrations greater than 3.5 μg/L were defined as elevated since previous research indicates this may be a more appropriate safety threshold for women during pregnancy than the threshold established for adults in general [24].

### 2.4. Data Analysis

Given the ordinal nature of the mercury categories, we used cumulative logistic models to assess the relationships between blood mercury levels and race, age, education, household income, and marital status. These cumulative logistic models relied on the proportional odds assumption, which implies that the effects of the independent covariates are the same across logits. Score tests were used to check for violations of this assumption, and in all analyses reported here, this test supported the validity of this assumption.

Based on the dietary questionnaire, we constructed five variables to characterize fish consumption habits. Three dichotomous variables for eats canned tuna, eats fish bought at a store or restaurant, and eats fish caught by family or friends were merged into a fourth dichotomous variable indicating whether the participant reported any fish consumption. A fifth variable summed the reported servings of fish from each source into a continuous variable representing the total fish meals per week. Using logistic models for the dichotomous measures and linear regression for the continuous measure, we examined the relationships between fish consumption habits and maternal demographic and socioeconomic characteristics. We then used cumulative logistic models to assess the relationships between blood mercury levels and each measure of fish consumption.

Finally, we modeled maternal blood mercury level as a function of demographics, socioeconomic status, and fish consumption habits. We fit three versions of this multivariate cumulative logistic model, one which included the dichotomous measure of eats fish from any source, one which included three dichotomous variables indicating fish consumption from each source (canned tuna, store/restaurant-bought fish, and caught fish), and one which included the continuous measure of number of fish meals per week. All analyses were undertaken using SAS 9.2 (SAS Institute, Cary, NC).

## 3. Results

The analyses presented in this paper were performed on data for women enrolled between study inception (June 2005) and December 2008 and delivered by the end of March 2009. We restricted the analysis to participants who completed the study and for whom a valid blood mercury level was reported from Mayo Medical Laboratories. Of the 1,209 women enrolled in the study and expected to deliver by the end of March 2009, 2.2% withdrew from the study and 6.5% were lost to follow-up. In addition, we excluded 84 women who did not provide a blood sample and 77 women whose samples were not able to be analyzed due to technical issues. Excluded participants did not differ from participants included in this analysis in terms of demographic or socioeconomic characteristics. The final sample consisted of 927 participants. Due to missing data on income or dietary choices, the number of observations available for different aspects of this analysis varied.

Our study population consisted predominantly of minority, unmarried, low-income women. The population was over two-thirds non-Hispanic black (NHB), just under one-fifth non-Hispanic white (NHW), and roughly 5% each of Hispanic (H) and Asian/Pacific Islander (A/PI), with about 2% of the population reporting multiple or other races. This oversampling of NHB was intentional in the design of the Healthy Pregnancy, Healthy Baby Study. Most of our participants were 20–34 years of age (71.4%), and most were not married (64.8%). Half the participants (50.9%) had only a high school education or less, and 53.3% of those reporting a yearly household income lived in households with an income of less than $20,000.

### 3.1. Mercury Levels

Overall, 69.7% of participants had a blood mercury level less than the detection limit of 1 μg/L, 28.4% had a detectable blood mercury level that was less than the safety threshold for pregnant women (3.5 μg/L), and 1.9% had a blood mercury level classified as elevated (*i.e.*, above the level of concern for pregnant women). The maximum blood mercury level in the study population was 8 μg/L. Eight women (0.9%) had a blood mercury level higher than US Environmental Protection Agency’s safety threshold for adults (5.8 μg/L). Although two-thirds of our population had blood mercury levels below the detection limit of 1 μg/L, previous studies have demonstrated adverse health effects of maternal mercury exposure at levels below 1 μg/L [11]. Unfortunately, data limitations prevent us from exploring associations between low mercury levels and mercury sources.

Table 1 presents the distribution of blood mercury levels within each demographic and socioeconomic subgroup, as well as the results of cumulative logistic models assessing the associations between each of these maternal characteristics and blood mercury levels. Race, age, education, household income, and marital status were each associated with blood mercury level (all p < 0.01). Blood mercury levels were higher among A/PI women compared to all other race groups. The probability of having a higher blood mercury level increased with age, and unmarried women were significantly more likely to have a higher blood mercury level than married women. More socioeconomically advantaged women were also at higher risk of higher blood mercury levels, with the likelihood of having a higher blood mercury level increasing with household income and educational attainment beyond the high school level.

### 3.2. Fish Consumption

Information on fish consumption habits was available for 867 participants. Overall, about two-thirds of the participants reported eating fish from at least one source, with over half reporting consumption of store/restaurant-bought fish, and only 10% eating fish caught by someone they knew. Table 2 summarizes the fish consumption habits of participants by demographic and socioeconomic characteristics.

Eating fish from any of the three sources was only significantly associated with age, with women 35 years and older being more likely to report fish consumption from at least one source than younger women (p < 0.05). Reported consumption of canned tuna was not significantly associated with any demographic or socioeconomic characteristic.

Consumption of store/restaurant-bought fish was associated with race, age, household income, and marital status (all p < 0.05). Over three-fourths of A/PI women reported consumption of store/restaurant-bought fish, making them significantly more likely to report consumption from this source than women of all other race groups. H women were less likely to report eating store/restaurant-bought fish than NHB women (42.1% *vs.* 56.7%, respectively), with NHB and NHW showing comparable consumption levels. Among women aged 18–19 years, only 43.6% of women reported consuming fish bought at a store or restaurant, a significantly lower rate than that seen among women 20 years of age and older. Women in the highest household income group were also significantly more likely to eat store/restaurant-bought fish than those in the lowest income group, and unmarried women were less likely to eat store/restaurant-bought fish than married women.

Race, education, and household income were associated with reporting consumption of fish caught by someone known to the participant (all p < 0.05). Compared to NHW women, NHB and H women were over 3 times as likely to eat fish caught by themselves or a friend/family member. About 7% of women with more than a high school education reported eating fish caught by someone they knew, while significantly higher rates of consumption of caught fish were reported by women with lower educational attainment (∼14%). Women in the highest household income category were half as likely to eat fish caught by someone they knew compared to the lowest income group.

The continuous measure of fish consumption (number of fish meals per week) was associated with race, education, household income, and marital status (all p < 0.05). On average, NHB women reported eating one additional fish meal per week compared to NHW women (p < 0.05). The mean number of fish meals per week was also significantly higher among women who completed high school compared to women who had a higher educational attainment (difference = 0.7, 95% CI = 0.2–1.1) and among women in the lowest household income group compared to women in the highest household income group (difference = 0.7, 95% CI = 0.2–1.2). Unmarried women reported eating an average of 1.8 fish meals per week, while married women reported eating an average of 1.5 fish meals per week (p < 0.05).

Table 3 presents the distribution of mercury levels among women reporting consuming fish from any source, canned tuna, fish from a store or restaurant, and fish caught by someone known to them. The odds ratios (OR) presented in the table indicate the relative odds of being in a higher blood mercury level when fish consumption is reported compared to when fish consumption is not reported. Since the proportional odds assumption was not violated, these OR represent comparisons of both being in the elevated *versus* detectable or below detection categories and being in the elevated or detectable *versus* below detection categories. As expected, being in a higher blood mercury category was associated with consumption of fish from any source, canned tuna, and store/restaurant-bought fish (all p < 0.05). Although only about 10% of women reported eating fish caught by someone they knew, we still found a marginally significant association between higher blood mercury levels and eating caught fish (p = 0.08). In a cumulative logistic model for blood mercury level that included an indicator for each fish source, the odds of being in a higher blood mercury category increasing by 42% when consumption of canned tuna was reported (OR = 1.42, 95% CI = 1.00–1.94) and by 165% when consumption of store/restaurant-bought fish was reported (OR = 2.65, 95% CI = 1.92–3.65). Using the continuous measure of total fish meals per week, each additional fish meal was associated with a 6% increase in the odds of a higher blood mercury level (OR = 1.06; 95% CI = 1.01–1.12).

### 3.3. Multivariate Analysis

In order to more fully explore the relationship between blood mercury levels and maternal characteristics, we modeled the ordered 3-category blood mercury level using cumulative logistic models controlling for race, age, education, household income, marital status, and dietary habits. We fit three versions of this multivariate cumulative logistic models, one which included the dichotomous measure of fish consumption from any source (Model 1), one which included the three dichotomous variables indicating fish consumption from each source (canned tuna, store/restaurant-bought fish, and caught fish) (Model 2), and one which included the continuous measure of number of fish meals per week (Model 3). Table 4 presents the results of each of these multivariate cumulative logistic models.

Although significant in univariate analyses, education, household income, and marital status were not significantly related to blood mercury levels when simultaneously controlling for all covariates. Race and age remained associated with blood mercury levels in each of the three multivariate models (all p < 0.05), and these relationships followed similar patterns to those seen in the univariate analyses. The odds of having a higher blood mercury level was consistently 3–4 times higher for A/PI women compared to both NHW (see Table 4 for ORs) and NHB women (Model 1: OR = 3.27, 95% CI = 1.50–7.12; Model 2: OR = 3.35, 95% CI = 1.50–7.48; Model 3: OR = 3.65, 95% CI = 1.66–8.03). Women aged 20 years and older were significantly more likely to have a higher blood mercury level than women aged 18–19 years (p < 0.05, except p = 0.06 for 20–34 years *versus* 18–19 years in Model 2), however there was no significant difference between women 20–34 years and those aged 35 years and older.

While controlling for demographic and socioeconomic characteristics, the expected relationships between blood mercury levels and fish consumption habits remained strong. Reporting fish consumption from any source (Model 1) more than doubled the odds of having a higher blood mercury level (OR = 2.56, 95% CI = 1.75–3.74), almost identical to the association seen when not controlling for covariates. In Model 2, consumption of canned tuna and store/restaurant-bought fish were significantly associated with higher blood mercury levels in patterns similar to the model that did not include maternal covariates, with the odds of having a higher blood mercury level increasing by 68% when consumption of canned tuna was reported (OR = 1.68, 95% CI = 1.70–3.51) and by 144% when consumption of store/restaurant-bought was reported (OR = 2.44, 95% CI = 1.70–3.51). When fish consumption habits were entered into the multivariate model as a continuous measure, each additional fish meal per week was associated with a 10% increase in the odds of a higher blood mercury level (OR = 1.10; 95% CI = 1.03–1.17).

## 4. Discussion

Previous work indicates that fish consumption and mercury levels vary across geography and demographics. Data from the 1999–2004 National Health and Nutrition Examination Survey (NHANES) revealed that blood mercury levels were highest among US women of childbearing age living in the Northeast and lowest among those living in the Midwest [29]. In a study of pregnant women in New Jersey, Stern found that 92% of his sample reported some fish consumption, with canned tuna the most commonly consumed fish. Consumption of caught fish was reported by 23% of the sample. The range of mercury in blood was less than 0.5 to 32 μg/L, with 15% having mercury levels above the detection limit of 0.5 μg/L and 4.7% having levels above 5 μg/L. The lowest mercury levels were found among blacks and those with some college [31]. A maternal and child health study in Michigan reported that older age, being white or “other” race, and not being insured by Medicaid were associated with higher maternal mercury levels after adjusting for fish consumption [3]. A Wisconsin Division of Public Health report indicated that higher mercury levels were associated with higher socioeconomic status [32].

This analysis describes fish consumption and mercury levels in a predominantly NHB, urban pregnant population in a noncoastal area of the US South. Overall, our results are similar to previous US studies in that the blood mercury levels of the participants were relatively low [4,31,33]. It is unclear, though, whether there is any safe level of mercury exposure during pregnancy, so even the low levels reported here may be of concern.

Sample demographics predicted the source of the fish consumed by participants. Two-thirds of the sample reported eating fish from at least one source, with older women more likely to consume fish than younger women. Consumption of canned tuna did not vary by demographic characteristics, and, in contrast to the study in New Jersey [31], store/restaurant-bought fish rather than canned tuna was the most common source of fish reported by our participants. Demographic distribution was as expected for store/restaurant-bought fish, with older, married, better educated, and higher income women most likely to report consumption of store/restaurant-bought fish. Asian/Pacific Islander women were over twice as likely as all other women to report consumption of store/restaurant-bought fish, and Hispanic women were the least likely to report consumption from this source. Trends were the reverse for reported consumption of caught fish. Women who were more likely to report caught fish consumption were Hispanic, younger, had completed less than a high school education, had lower incomes, and were not married. However, when looking at total fish meals, non-Hispanic black, lower educated, unmarried women with lower incomes consumed a greater number of fish meals per week.

Almost a third of our sample had a detectable blood mercury level, and about 2% had blood mercury levels above the level of concern for pregnant women. Compared to socioeconomically-advantaged women, socioeconomically-disadvantaged women were at a reduced risk of having a high blood mercury level, despite the fact that, on average, they were consuming more fish meals per week. Consistent with the previous studies in the US [29,32], mercury levels in this population were higher among Asian/Pacific Islander, older, higher educated, higher income, and married women.

As expected, higher blood mercury levels were associated with fish consumption from any source. Consumption of canned tuna and store/restaurant-bought fish increased the odds of having a higher blood mercury level. In addition, each additional fish meal slightly increased the odds a higher blood mercury level. The low rate of consumption of caught fish in our sample may have reduced our power to detect an association between caught fish and mercury levels. Importantly, North Carolina has a statewide mercury fish advisory for largemouth bass, a popular fish among recreational anglers [34].

An important contribution of our work is that lower income, lower educated women in our study report eating more fish meals than the higher income, higher educated women, but have lower mercury levels. The federally-funded Women, Infants and Children (WIC) supplemental food program is designed to meet the nutritional needs of low-income pregnant, breastfeeding, and postpartum women, as well as their infants and children up to age 5. WIC disperses vouchers for canned chunk light tuna, salmon, sardines, or mackerel as part of its meal supplementation plan. Salmon has high concentrations of omega-3 fatty acids and low mercury levels (1.1–1.9 g omega-3 fatty acids and 0.01 ppm mercury per 3 ounce serving). The canned tuna covered by WIC vouchers has lower omega-3 fatty acid levels and moderate concentrations of mercury (0.17–0.24 g omega-3 fatty acids and 0.12 ppm mercury per 3 ounce serving) compared to salmon, but is notably lower in mercury content than most other types of fish [35,36]. Because our population is primarily of low socioeconomic status, this may be an explanation for why our lower income, lower educated women have lower mercury levels despite eating more fish meals per week. These results suggest that directing women’s fish consumption, at least through the incentives built into the WIC program, can successfully increase fish consumption without necessarily increasing mercury exposure.

Our sample of over 900 women, which is quite large compared to other studies of blood mercury levels, is predominantly non-Hispanic black and low-income, with relatively few Hispanics due to our inclusion criteria of being English-literate. Similarly, there are relatively few Asian/Pacific Islanders in the sample. Our results are thus likely more generalizable for NHB than for other racial and ethnic groups. Importantly, however, NHB are traditionally understudied with respect to mercury exposure. Our fish consumption questions are general, limiting our ability to delineate between types of fish consumed. In addition, the self-reported question is based on a typical week, reflecting average consumption. We also measure total blood mercury levels rather than the more appropriate methylmercury levels. Despite these limitations, this study provides a description of fish consumption and blood mercury levels in a large prospective cohort study in an urban pregnant population in the American South.

## 5. Conclusions

The adverse effects of mercury have been detected even at very low exposure levels. Based on NHANES data from 1999–2006, approximately half of the women of childbearing age in the United States had at least 1 μg/L mercury in their blood [33]. The median decreased slightly from 2001–2004 but increased again in 2005–2006. In our study population, from an inland area in the US South, over 30% of women had at least 1 μg/L mercury in their blood. Cord blood levels of mercury are on average 70% higher than maternal blood mercury levels [37]. Moreover, ongoing neurodevelopment in an infant’s brain throughout the first year creates critical post-natal vulnerabilities to methylmercury exposure [38].

Pregnant women should be encouraged to consume fish but to choose their fish wisely in order to lower the risk of mercury exposure. The higher mercury levels among women of higher socioeconomic status indicate that current messages regarding how to make fish choices wisely during pregnancy may not be reaching higher income groups. In contrast, women enrolled in WIC, whose fish consumption choices are in many ways directed by the incentives within the program to consume high-omega-3/low mercury fish, consumed the greatest number of fish meals per week and had the lowest mercury levels. This suggests that coupling incentives with messaging may be an effective approach to managing mercury exposure during pregnancy.

The messages regarding fish consumption, especially during pregnancy, are complex. Fish consumption is good for fetal development; mercury exposure is not. Relaying this complex health message can be challenging and must be done with cultural sensitivity and awareness, which can only be accomplished by improving our knowledge of fish consumption patterns and the distribution of blood mercury levels among diverse populations.

## Figures and Tables

**Table 1.** Distribution of blood mercury levels by demographic and socioeconomic characteristics and results from simple cumulative logistic models for mercury levels.

			**Mercury level 1**			**Cumulative logistic models 2**	

		**All**	**Below detection limit**	**Detectable, not elevated**	**Detectable, elevated**	**Type III**	**Groups 3**
*Overall*	N	927	646	263	18		
	row %		69.7%	28.4%	1.9%		

*Maternal race/ethnicity*						<0.01	
Non-Hispanic white	N	181	122	56	3		A
	row %		67.4%	30.9%	1.7%		
Non-Hispanic black	N	629	455	165	9		A
	row %		72.3%	26.2%	1.4%		
Hispanic	N	58	40	17	1		A
	row %		69.0%	29.3%	1.7%		
Asian/Pacific Islander	N	40	13	22	5		
	row %		32.5%	55.0%	12.5%		

*Maternal age*						<0.01	
18–19 years	N	134	109	23	2		
	row %		81.3%	17.2%	1.5%		
20–34 years	N	662	472	178	12		
	row %		71.3%	26.9%	1.8%		
≥35 years	N	131	65	62	4		
	row %		49.6%	47.3%	3.1%		

*Maternal education*						<0.01	
Less than high school	N	114	86	26	2		B
	row %		75.4%	22.8%	1.8%		
Completed high school	N	358	276	78	4		B
	row %		77.1%	21.8%	1.1%		
Beyond high school	N	455	284	159	12		
	row %		62.4%	34.9%	2.6%		

*Yearly household income*						<0.01	
<$20, 000	N	441	341	96	4		
	row %		77.3%	21.8%	0.9%		
$20,000–$39,999	N	169	118	47	4		
	row %		69.8%	27.8%	2.4%		
≥$40,000	N	217	122	88	7		
	row %		56.2%	40.6%	3.2%		

*Marital status*						<0.01	
Married	N	326	192	123	11		
	row %		58.9%	37.7%	3.4%		
Not married	N	599	452	140	7		
	row %		75.5%		23.4%	1.2%	

1 Maternal blood mercury level categories are defined as: below the detection limit (<1 μg/L), detectable but not elevated (1–3 μg/L), and elevated (≥4 μg/L).

2 A separate cumulative logistic model for mercury level was fit for each covariate. Mercury level was defined as (1) below the detection limit, (2) detectable but not elevated, and (3) detectable and elevated. Score tests in each model indicated that the proportional odds assumption was not violated (p > 0.05).

3 Levels of each covariates identified with the same letter were not significantly different at α = 0.05.

**Table 2.** Percent reporting fish consumption from various sources (among those answering each survey question) and mean total reported fish meals per week by demographic and socioeconomic characteristics.

	**Any source**		**Canned tuna**	**Store/restaurant bought fish**		**Caught fish**		**Total fish meals**		
								**Mean**	**SD**	
*All*	67.8%		35.8%	56.3%		10.3%		1.7	2.5	

*Maternal race/ethnicity*					**		*			**
Non-Hispanic white	67.6%		35.2%	56.7%		3.9%		1.0	1.1	
Non-Hispanic black	68.0%		36.6%	56.0%		12.1%		2.0	2.9	
Hispanic	59.7%		37.5%	42.1%		14.3%		1.3	1.8	
Asian/Pacific Islander	79.0%		23.7%	79.0%		5.4%		1.4	1.6	

*Maternal age*		*			**					
18–19 years	60.5%		31.5%	43.6%		12.1%		1.6	2.6	
20–34 years	67.6%		35.6%	56.9%		10.1%		1.7	2.5	
≥35 years	76.0%		40.6%	65.9%		9.4%		1.7	2.4	

*Maternal education*							**			**
Less than high school	63.9%		39.1%	48.6%		14.3%		2.0	2.9	
Completed high school	68.2%		38.0%	55.1%		13.8%		2.0	2.8	
Beyond high school	68.6%		33.3%	59.1%		6.7%		1.4	2.0	

*Yearly household income*					**		*			**
<$20,000	64.5%		37.5%	51.4%		12.6%		1.9	2.9	
$20,000–$39,999	72.7%		30.9%	58.9%		7.4%		1.7	2.6	
≥$40,000	70.4%		34.3%	64.6%		5.7%		1.2	1.3	

*Marital status*					**					*
Married	71.5%		36.3%	65.9%		7.6%		1.5	1.9	
Not married	65.7%	35.6%		52.5%		11.8%		1.8	2.8	

** p < 0.01,

* p < 0.05 for an association between fish consumption measures and the demographic or socioeconomic characteristic.

**Table 3.** Distribution of mercury levels among participants reporting fish consumption from various sources.

			**Mercury Level 1**			**Cumulative logistic model 2**	

		**All**	**Below detection limit**	**Detectable, not elevated**	**Detectable, elevated**	**OR**	**95% CI**
*Any source*	N	601	381	205	15	2.56	(1.82, 3.60)
	row % 3		63.4%	34.1%	2.5%		

*Canned tuna*	N	313	194	114	5	1.68	(1.25, 2.26)
	row %		62.0%	36.4%	1.6%		

*Store/restaurant-bought fish*	N	496	298	184	14	2.87	(2.10, 3.92)
	row %		60.1%	37.1%	2.8%		

*Caught fish*	N	89	55	31	3	1.51	(0.96, 2.36)
	row %		61.8%	34.8%	3.4%		

1 Maternal blood mercury level categories are defined as: below the detection limit (<1 μg/L), detectable but not elevated (1–3 μg/L), and elevated (≥4 μg/L).

2 A separate cumulative logistic model for mercury level was fit for each measure of fish consumption. The OR indicates the relative odds of being in a higher mercury category when mercury level was defined as (1) below the detection limit, (2) detectable but not elevated, and (3) detectable and elevated. Score tests in each model indicated that the proportional odds assumption was not violated (p > 0.05).

3 Row percents are among those who reported consumption from the specified source, not among those who responded to the survey question.

**Table 4.** Covariate-adjusted odds ratios and 95% confidence intervals from multivariate cumulative logistic models for blood mercury level.

	**Model 1: fish consumption from any source (n=799)**		**Model 2: fish consumption by source (n=781)**		**Model 3: total fish meals per week (n=776)**	

	**aOR**	**95% CI**	**aOR**	**95% CI**	**aOR**	**95% CI**
*Maternal race/ethnicity*						
Non-Hispanic white	1.0	—	1.0	—	1.0	—
Non-Hispanic black	1.27	(0.80, 2.02)	1.29	(0.80, 2.07)	1.22	(0.76, 1.94)
Hispanic	1.59	(0.72, 3.51)	1.68	(0.75, 3.75)	1.52	(0.70, 3.31)
Asian/Pacific Islander	4.16	(1.93, 8.94)	4.31	(1.96, 9.49)	4.44	(2.04, 9.68)

*Maternal age*						
18–19 years	1.0	—	1.0	—	1.0	—
20–34 years	2.07	(1.11, 3.86)	1.85	(0.98, 3.48)	2.03	(1.09, 3.79)
≥35 years	3.22	(1.54, 6.74)	2.81	(1.32, 5.97)	3.14	(1.49, 6.62)

*Maternal education*						
Less than high school	1.12	(0.62, 2.00)	1.16	(0.63, 2.11)	1.10	(0.60, 2.00)
Completed high school	1.0	—	1.0	—	1.0	—
Beyond high school	1.29	(0.85, 1.95)	1.38	(0.90, 2.12)	1.37	(0.90, 2.08)

*Yearly household income*						
<$20,000	0.81	(0.52, 1.26)	0.73	(0.47, 1.15)	0.77	(0.49, 1.20)
$20,000–$39,999	1.0	—	1.0	—	1.0	—
≥$40,000	1.25	(0.74, 2.10)	1.18	(0.69, 2.00)	1.27	(0.75, 2.13)

*Marital status*						
Married	1.0	—	1.0	—	1.0	—
Not married	0.82	(0.53, 1.25)	0.86	(0.55, 1.33)	0.81	(0.53, 1.25)

*Fish consumption*						
Any source	2.56	(1.75, 3.74)				
Canned tuna			1.68	(1.19, 2.38)		
Store/restaurant-bought fish			2.44	(1.70, 3.51)		
Caught fish			1.04	(0.60, 1.80)		
Total fish meals per week					1.10	(1.03, 1.17)
